# Phytochemical Profile, α-Glucosidase, and α-Amylase Inhibition Potential and Toxicity Evaluation of Extracts from *Citrus aurantium* (L) Peel, a Valuable By-Product from Northeastern Morocco

**DOI:** 10.3390/biom11111555

**Published:** 2021-10-20

**Authors:** Ouijdane Benayad, Mohamed Bouhrim, Salima Tiji, Loubna Kharchoufa, Mohamed Addi, Samantha Drouet, Christophe Hano, Jose Manuel Lorenzo, Hasnae Bendaha, Mohamed Bnouham, Mostafa Mimouni

**Affiliations:** 1 Laboratory of Applied Chemistry and Environment (LCAE), Faculty of Sciences Oujda (FSO), University Mohammed First (UMP), Oujda 60000, Morocco; salimatiji@gmail.com (S.T.); hasnae.bendaha@gmail.com (H.B.); mimouniosrn@gmail.com (M.M.); 2 Laboratory of Bioresources, Biotechnology, Ethnopharmacology and Health, Faculty of Sciences Oujda (FSO), University Mohammed First (UMP), Oujda 60000, Morocco; mohamed.bouhrim@gmail.com (M.B.); Kharchoufa.loubna@ump.ac.ma (L.K.); mbnou-ham@yahoo.fr (M.B.); 3 Laboratoire dʼAmélioration des Productions Agricoles, Biotechnologie et Environnement (LAPABE), Faculté des Sciences, Université Mohammed Premier, Oujda 60000, Morocco; m.addi@ump.ac.ma; 4 Laboratoire de Biologie des Ligneux et des Grandes Cultures, INRA USC1328, Orleans University, CEDEX 2, 45067 Orléans, France; samantha.drouet@univ-orleans.fr; 5 Centro Tecnológico de la Carne de Galicia, Adva. Galicia n° 4, Parque Tecnológico de Galicia, San Cibrao das Viñas, 32900 Ourense, Spain; 6 Área de Tecnología de los Alimentos, Facultad de Ciencias de Ourense, Universidad de Vigo, 32004 Ourense, Spain

**Keywords:** *Citrus aurantium* peel, by-product, sequential extraction, bioactive phytochemicals, α-glucosidase, α-amylase, mice toxicity

## Abstract

Due to the high volume of peel produced, *Citrus* by-product processing could be a significant source of phenolic compounds, in addition to essential oil. *Citrus* fruit residues, which are usually dumped as waste in the environment, could be used as a source of nutraceuticals. *Citrus aurantium* (L), also known as sour or bitter orange, is a member of the Rutaceae family and is the result of interspecific hybridization between *Citrus reticulata* and *Citrus maxima*. The purpose of this study is to chemically and biologically evaluate the peel of *C. aurantium*, which is considered a solid waste destined for abandonment. To achieve more complete extraction of the phytochemicals, we used a sequential extraction process with Soxhlet using the increasing polarity of solvents (i.e., cyclohexane, chloroform, ethyl acetate, acetone, and ethanol–water mixture). Essential oil (EO) from the *Citrus* peel, which was present at 1.12%, was also prepared by hydrodistillation for comparison. Various phytochemical assays were used to determine the qualitative chemical composition, which was subsequently characterized using GC-MS and HPLC-DAD. The inhibitory effects of *C. aurantium* peel extract on two enzymes, intestinal α-glucosidase and pancreatic α-amylase, were measured in vitro to determine their potential hypoglycemic and antidiabetic actions. Each extract had a significantly different phytochemical composition. According to GC-MS analyses, which allow the identification of 19 compounds, d-limonene is the most abundant compound in both EO and cyclohexane extract, at 35.17% and 36.15% (*w*/*w*). This comparison with hydrodistillation shows the value of the sequential process in extracting this valuable terpene in large quantities while also allowing for the subsequent extraction of other bioactive substances. On the contrary, linoleic acid is abundant (54.35% (*w*/*w*)) in ethyl acetate extract (EAE) with a lower amount of d-limonene. HPLC-DAD analysis allows the identification of 11 phytochemicals, with naringenin being the most abundant flavanone, detected in acetone extract (ACE) (23.94% (*w*/*w*)), ethanol–water extract mixture (EWE) (28.71% (*w*/*w*)), and chloroform extract (CFE) (30.20% (*w*/*w*)). Several extracts significantly inhibited α-amylase and/or α-glycosidase in vitro. At a dose of 332 g/mL, ACE, CFE, and EWE inhibited the two enzymes by approximately 98%. There were strong significant correlations between naringenin and α-glucosidase inhibition and between gallic acid and α-amylase inhibition. Molecular docking experiments further verified this. Finally, oral administration of *C. aurantium* extracts at a dose of 2000 mg/kg did not cause any effect on mice mortality or signs of acute toxicity, indicating that it is non-toxic at these doses. These findings suggest that *C. aurantium* peels could be a valuable by-product by providing a rich source of non-toxic phytoconstituents, particularly those with potential antidiabetic action that needs to be confirmed in vivo.

## 1. Introduction

*Citrus aurantium*, also known as sour orange, is native to Southern to Eastern Asia, Malaysia, New Caledonia, and Australia [1]. It was later cultivated in Spain, France, North and South Africa, and the rest of the tropical to the temperate world because it is the most resistant plant of all *Citrus* species [2].

*Citrus* production has increased in recent years, and the fruit processing industries have focused on the production of juices, essential oils, flavoring, antioxidants, and acidifying agents for food [3,4]. However, the amount of solid *Citrus* waste (peels, seeds, and membrane residue) generated after processing is huge. Several studies on the recycling and valorization of solid *Citrus* waste have revealed a wide range of applications, particularly as a source of phytochemicals vitamins and/or minerals for the treatment of various diseases [5,6]. The main bioactive constituents of *C. aurantium* are flavonoids, which have been shown to have antioxidant [7,8], antimicrobial, antiallergic, anticancer, and antidiabetic properties [9]. Furthermore, *C. aurantium* has been used in herbal medicine as a stimulant and appetite suppressant; it has also been used in traditional Chinese medicine to treat nausea, indigestion, and constipation. It is also used to treat cancer and certain cardiovascular diseases [10].

Diabetes mellitus, a serious disease that can be defined as a group of metabolic disorders characterized by chronic hyperglycemia, is common throughout the world. Diabetes is classified into two types: type 1 (insulin-dependent diabetes), which occurs as a result of the pancreas’ inability to secrete enough insulin due to beta cell destruction, and type 2 (non-insulin-dependent diabetes) [11]. Cells in type 2 (non-insulin-dependent) diabetes no longer respond to insulin and become insulin resistant. There are currently several synthetic drugs used to treat diabetic patients; the main goal is to reduce postprandial hyperglycemia by inhibiting digestive enzymes, primarily intestinal α-glucosidase and pancreatic α-amylase, or by reducing glucose absorption by the intestine. Several plant natural products/extracts have emerged as promising α-glucosidase and α-amylase inhibitors in recent decades [12,13,14,15,16]. Furthermore, recent research has emphasized the importance of promoting safer and tolerable inhibitors for the two enzymes that are naturally extracted from medicinal plants, fruits, and vegetables at a lower cost, particularly *Citrus* fruits. Many studies have shown that their consumption helps to treat a variety of chronic diseases, including type 2 diabetes [17]. It has been reported that navel orange contains significant antidiabetic constituents. Furthermore, another study on some selected *Citrus* species from Jordan revealed potential α-glucosidase and α-amylase inhibitory activities [18].

The goal of this study was to extract and separate secondary metabolites from the *C. aurantium* peel using the polarity of solvents, moving from a non-polar solvent (cyclohexane) to a more polar solvent mixture (ethanol–water). Here, these sequential extraction methods take advantage of the fact that different solvents have different polarities and thus different extraction capacities for different compounds. *Citrus* peel was therefore extracted using a series of solvents, each selected to selectively extract a single or a group of compounds with similar chemical properties. It was supported by both qualitative and quantitative phytochemical analyses, followed by GC-MS and HPLC-DAD characterization and identification of the compounds in comparison to local database abs standards. This research focused on the potential antidiabetic activity of α-glucosidase and α-amylase enzyme inhibition. A systematic study of the in vivo toxicity of all extracts on mice was performed.

## 2. Materials and Methods

### 2.1. Chemicals

All solvents (cyclohexane, chloroform, ethyl acetate, acetone, ethanol, dimethyl sulfoxide (DMSO)), analytical grade (99.5%), were purchased from Sigma-Aldrich. Acarbose was purchased from Bayer Schering Pharma. Intestinal α-glucosidase type I (10 units/mg of proteins), pancreatic α-amylase, and all the reagents were purchased from Sigma-Aldrich.

### 2.2. Plant Material

Sour oranges were collected locally from an average of 10 *C. aurantium* trees, for a good sampling, grown in Eastern Morocco, in the period between November and February. The fruits were washed in running water and then in distilled water, and they were peeled. The peels were divided into two parts; the first one was used fresh for the extraction of essential oil, while the second was dried on the stove, for 3 days, at a temperature of 35 to 45 °C and then ground into a fine powder using Moulinex LM242, a powerful grinder, to avoid any chemical degradation of the starting compounds due to the heat caused during the grinding process, which would further affect the test results.

### 2.3. Sample Preparation

*C. aurantium* peel essential oil was obtained by submitting 250 g of fresh peels to hydrodistillation for 3 h using the Clevenger type-apparatus. The essential oil was dried over anhydrous magnesium sulfate and conserved in dark flacons at 3 °C.

*C. aurantium* peel extracts were obtained by extracting, successively, 80 g of dried peel powder with 800 mL of solvents in increasing order of polarity (cyclohexane, chloroform, ethyl acetate, acetone, and ethanol–water) using the Soxhlet-type apparatus. The extracts were concentrated using a rotary vacuum evaporator and stored, away from light, at 3 °C for further use.

### 2.4. Phytochemical Investigation of C. aurantium Peel Extracts

Qualitative and quantitative phytochemical screening was performed for *C. aurantium* peel extracts to determine the nature of the families of chemical compounds present in each extract and the phenol and flavonoid total content as well.

#### 2.4.1. Qualitative Screening

A screening of steroids/terpenoids, alkaloids, flavonoids, saponins, phenols and tannins, coumarins, and free quinone was performed based on the color intensity or precipitate formation that are considered analytical responses to these tests.

The Liebermann–Burchard test was used by adding 2 mL of chloroform to each extract and then acetic anhydride, and concentrated H_2_SO_4_, and the color of the mixture turned to red, blue, and then green, which indicated the presence of steroids and terpenoids [19].

The extracts were recuperated in a few milliliters of HCl (50%), to which then Mayer reagent was added. The appearance of a white or yellow precipitate indicated the the entity of alkaloids [20].

Exactly 0.5 g of magnesium ribbon and concentrated HCl were mixed with each extract. A pink-colored precipitate appeared that indicated the the presence of flavonoids [19].

A volume of 2 mL of distilled water was added to each extract in a test tube, and it was shaken vigorously. The foam formation indicated a positive test for the revelation of saponins [19].

A volume of 2 mL of 1% (*w*/*v*) solution of FeCl_3_ was mixed with crude extract, and a black or blue-green color indicated the presence of tannins and phenolics [19].

The extracts were put in test tubes, and they were covered with filter paper soaked with diluted NaOH. The tubes were placed in a water bath for a few minutes, and then the paper was examined under UV light. Yellow fluorescence indicated the presence of coumarins [20].

A few drops of NaOH (1/10) aqueous solution were mixed with the extracts in test tubes, and a yellow, red, or purple color developed, testifying to the presence of quinones [20].

#### 2.4.2. Quantitative Screening

The phenolic and flavonoid total content was determined only for the extracts showing a positive test for those entities.

The total phenol content of the extracts was estimated according to the method described in the literature [8,21]. Briefly, 1 mL of Folin–Ciocalteu reagent diluted at 10% was mixed with 200 μL of the extracts or gallic acid (standards calibration) or methanol (blank). After 5 min, 800 µL of Na_2_CO_3_ solution (7.5 g/L) was added to the mixtures, which were stood for 1 h before their absorbance measurement at 700 nm using a UV–VIS spectrophotometer. Results were expressed as gallic acid equivalent (GAE) in milligrams per 100 g of dry matter.

The total flavonoid content of the extracts was determined using the colorimetric aluminum chloride method [8]. Briefly, 1 mL of diluted extract in methanol (2 mg/mL) was mixed with 1 mL of 2% aluminum chloride solution prepared in methanol. The mixtures was left at room temperature for 10 min, and then absorbance was measured at 430 nm using a UV–VIS spectrophotometer. Results were expressed as quercetin equivalent (QE) in milligrams per 100 g of dry matter.

### 2.5. Gaz Chromatography Coupled with Mass Spectroscopy (GC-MS) Analysis

The essential oil from *C. aurantium* peel and the mother extracts of ethyl acetate and cyclohexane were analyzed using a Shimazadu QP 2010 GC-MS apparatus equipped with a DB-5 capillary column (30 m long, 0.25 mm diameter, 0.25 µm film thickness). Helium was used as a carrier gas with a flow rate of 1 mL/min. The initial temperature of the column was 60 °C. It was gradually increased to finally reach 210 °C with a step of 10 °C/min. Then, 1 μL of the diluted samples 1:100 (*v*/*v*) with hexane was injected in spitless mode. The temperature of the injector and detector was set at 250 °C and 280 °C, respectively.

Chemical compounds present in each sample were identified based on the GC retention time on the DB-5MS column and matching of the spectra with computer software data of standards.

### 2.6. High-Performance Liquid Chromatography Coupled with Diode Array Detector (HPLC-DAD) Analysis

*C. aurantium* peel chloroform, acetone, and ethanol–water extract analyses were performed using Waters 2695 Alliance Analytical HPLC equipped with a 2998 Photodiode Array detector. The column used was a C18 reverse-phase HPLC column (25 cm length, 4.6 mm diameter, and 5 μm particle size). The protocol used was according to that already described in the literature [22] with a slight modification, where the elution system was made up of two solvents: solvent A (acetonitrile) and solvent B (water with 2% (*v*/*v*) acetic acid glacial) at a flow rate of 0.9 mL/min. The initial condition gradient was 5% (*v*/*v*) A, which increased to 35% (*v*/*v*) A at 30 min and reached 70% (*v*/*v*) A at 45 min, to return to initial conditions, which were maintained for 5 min to equilibrate the column between analyses. Then, 20 μL of samples was injected and detected at λ = 280 nm and 350 nm.

Peak samples were identified and quantified by comparing their retention times and UV spectra in the chromatograms, respectively, with those of pure standards (vanillic acid, coumaric acid, gallic acid, caffeic acid, syringic acid, ascorbic acid, rosmarinic acid, p-coumaric acid, hydrobenzoic acid, chlorogenic acid, ferulic acid, kaempferol, quercetin, rutin, apigenin, catechin, tyrosol, naringenin, vanillin, malic acid, and citric acid) [23].

### 2.7. Inhibition Assay (In Vitro) for Intestinal α-Glucosidase Activity

Solutions of α-glucosidase (10 UI/mL), sucrose (50 mM), and glucose (1 g/L) were prepared by dissolving the enzyme, sucrose, and glucose, respectively, in phosphate buffer at pH 7.5. Meanwhile, all *C. aurantium* peel extracts and their essential oil were resuspended in DMSO and distilled water.

Intestinal α-glucosidase activity was evaluated by a spectrophotometer, following the release of glucose from sucrose, using a method described in the literature [24] with slight modifications. The quantity of liberated glucose was measured by the glucose oxidase–peroxidase (GOD-POD) method using a commercial test kit. In contrast, the inhibition test was examined for two concentrations of samples C_1_ (166 μg/mL) and C_2_ (332 μg/mL).

The assay mixtures contained 0.1 mL of sucrose (50 mM), 0.1 mL of α-glucosidase solution (10 UI/mL), 1 mL of phosphate buffer (50 mM) at pH 7.5, and 10 μL (166 μg/mL)/20 μL (332 μg/mL) of each sample. The volume of the sample was replaced by the same volume of distilled water, 20% DMSO, and Acarbose (166 μg/mL)/(332 μg/mL) for the control, negative control, and positive control, respectively.

The mixtures were incubated for 20 min at 37 °C, and the enzymatic reaction was stopped by heating for 5 min in a water bath at 100 °C. After adding 1 mL of GOD-POD, the mixtures were incubated again for 10 min at 37 °C. Finally, absorbance was measured at λ = 500 nm.

### 2.8. Inhibition Assay (In Vitro) for Pancreatic α-Amylase Activity

Solutions of α-amylase (13 UI/mL), substrate (soluble potato starch, 10 mg/mL) and samples (0.5 mg/mL, extracts, essential oil or Acarbose) were prepared in phosphate buffer at pH 6.9. Then 3,5-dinitrosalicylic chromogenic reagent (DNSA) was prepared as follows: 1 g of DNSA, 30 g of sodium potassium tartrate, and 20 mL of 2 N sodium hydroxide were adjusted to a final volume of 100 mL with distilled water [25].

Pancreatic α-amylase inhibitory assay was performed according to the method already described in the literature [26]. Briefly, 200 µL of plant sample solutions or Acarbose solution (positive control) or phosphate buffer solution (control) was added to 200 µL of the enzyme solution. The mixtures were pre-incubated for 10 min at 37 °C, and afterward, 200 µL of substrate solution was added, and the mixtures were incubated again for 15 min at 37 °C.

The enzymatic reaction was stopped by adding 600 µL of DNSA. The mixtures were placed in a water bath at 100 °C for 8 min to favor the reaction between DNSA and reducing sugars from starch hydrolysis. This reaction was stopped by a thermal shock, where the mixtures were placed in an ice-cold water bath, and then 1 mL of distilled water was added to dilute them and to facilitate the absorbance measurement at λ = 540 nm using a spectrophotometer.

In both cases of inhibition (α-amylase and α-glycosidase), the tests were carried out in three assays, and the percentage of inhibition was calculated according to the formula:

$$\mathrm{Inhibitory}\;\mathrm{activity}\;(\%)=\frac{Abs(control)-Abs(sample)}{Abs(control)}\times 100$$

where *Abs* (*control*) is the absorbance of the control mixture containing phosphate buffer and the enzyme and *Abs* (*sample*) is the absorbance of the sample mixtures containing plant samples or Acarbose and the enzyme.

### 2.9. Molecular Docking Analysis

PyRx virtual screening tool software, which includes Autodock 4 and Autodock Vina (Scripps Research Institute, La Jolla, CA, USA) and Pymol v2.1.1 (Schrodinger, New York, NY, USA), was used to predict the conformation of naringenin within the appropriate target-binding site of α-glucosidase (PDB: 5NN5). Discovery Studio 2020 (Dassault Systemes, Vélizy-Villacoublay, France) was used to determine the type of interaction and visualize it in 2D, while UCSF Chimera 1.14 (San Francisco, CA, USA) was used to represent molecules and interaction residues in 3D. The docking protocol employed was described by [16]. The ligand’s 3D structure was obtained from PubChem (available online: https://pubchem.ncbi.nlm.nih.gov/ (accessed on 25 August 2021)). To identify the most favorable binding site predicted by the program based on the lowest docking energy and the maximum docking number, an initial virtual screen with the entire enzyme was performed with a grid box of 81 Å × 82 Å × 85 Å (for α-glucosidase) in the x, y, and z dimensions, respectively. This site was then used to refine the docking with a grid box of 25 Å (square).

### 2.10. Acute Oral Toxicity of C. aurantium Samples

The toxic effect of *C. aurantium* peel samples was carried out on both sexes of animals, male and female albino mice, according to the Organization for Economic Co-operation and Development (OECD) guidelines [27]. The mice were cared for in compliance with the guidelines of the Declaration of Helsinki, and the study was approved by the institutional review board of the Faculty of Sciences, Oujda, Morocco (01/20-LBBEH-04 and 09/01/2020).

The healthy animals were divided into seven groups, with 3 males and 3 females per group. The first group (control) received distilled water orally, whereas the other groups (acute toxicity) received a single dose of peel samples (2000 mg/kg) body weight. Groups 1 to 6 received cyclohexane, chloroform, ethyl acetate, acetone, ethanol–water extracts, and EO of *C. aurantium* peel, respectively. Before oral administration, the animals were weighted and fasted overnight but with free access to water. Samples were administered to mice for 14 days.

The mice were observed individually for the first 30 min and then every hour for 6 h. Then, the animals were examined daily for any physiological changes (alteration, weight loss, damage to the skin or eyes) or general behavior (food intake, water consumption, respiration) or other dangerous symptoms.

### 2.11. Statistical Analysis

Data were presented as the mean ± standard error and were subjected to statistical analysis using Graph Pad Prism 5.04 software (San Diego, CA, USA) and XL-STAT (Addinsoft, Paris, France). Multiple-group comparisons were analyzed by one-way analysis of variance (ANOVA). Statistical significance was accepted as *p* ≤ 0.05.

## 3. Results

### 3.1. Yield of Extractions

**Figure 1:**
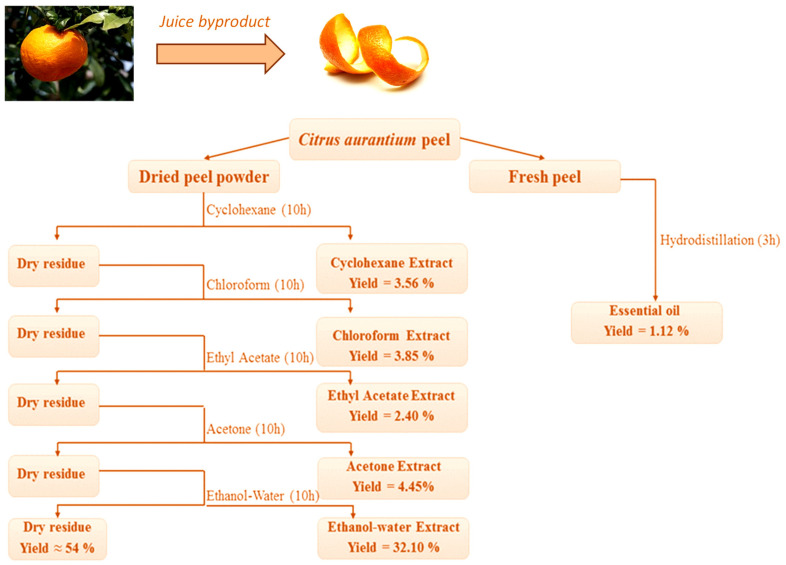
Extraction process and extraction yield by different solvents (cyclohexane, chloroform, ethyl acetate, acetone, and ethanol–water) from *C. aurantium* peel. Hydrodistillation of essential oil from the fresh peel.

Figure 1 depicts the extraction process from *C. aurantium* dried peel using polar and non-polar solvents, as well as the extraction yield. Due to its sensitivity to heat, only the essential oil was extracted from the fresh peel.

The total extraction yield was 46%, with the remainder consisting primarily of fibers and residues. Extraction with an ethanol–water mixture yielded the highest yield (32.10%), while extraction with ethyl acetate yielded the lowest (2.40%). The results show that the type of solvent used during the extraction process affects the yield. The yield of essential oil obtained from fresh peel (1.12%), however, is comparable to that found in the literature (ranging from 0.5% to 1.02%) [7,22,23].

### 3.2. Qualitative Phytochemical Screening

**Figure 2:**
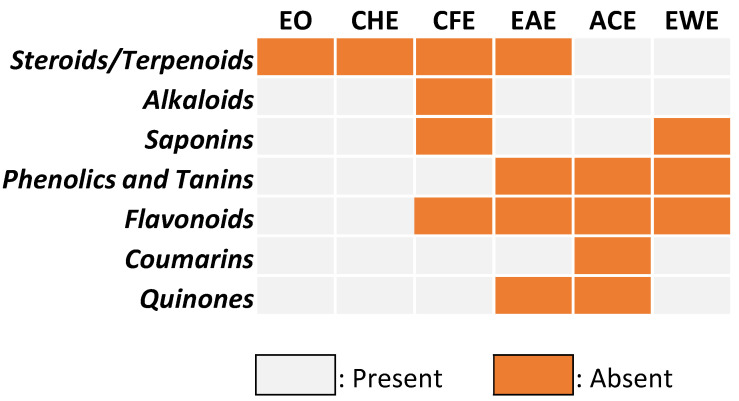
Qualitative phytochemical screening of *C. aurantium* peel extracts.

Figure 2 summarizes the secondary metabolites present in *C. aurantium* peel extracts for various solvents. These findings indicate that *Citrus* peel is an excellent source of phytochemicals that may detoxify free radicals by lowering oxidative stress [28]. Indeed, acetone extract was rich in flavonoids, phenolics, and tannins, whereas coumarins (polyphenolic compounds) were present in many extracts. Cyclohexane, chloroform, and ethyl acetate extracts were high in steroids and terpenoids, but only chloroform extract contained alkaloids. Surprisingly, quinone was only found in the extracts of ethyl acetate and acetone, whereas saponins were found in the extracts of chloroform and ethanol–water. Overall, our results are consistent with those published in the literature in many countries [7,29,30].

It should be noted that the extraction solvent composition is critical for extracting and separating specific secondary metabolites. Indeed, the polarity of the solvent is critical in determining the family of compounds to be extracted. For example, polar solvents are best for extracting phenolic compounds and flavonoids, whereas non-polar solvents are best for extracting steroids and terpenoids. Solvents with intermediate polarity can extract both types of families, which is why it is necessary to select suitable solvents with large polarity gaps to extract and separate the various families of compounds contained in the matrix at the same time. For example, we can sequentially extract and separate this matrix that constitutes *C. aurantium* peels using three solvents: cyclohexane (which extracts only steroids and terpenoids), acetone (which extracts flavonoids, phenolics, coumarins, and quinone), and an ethanol–water mixture (which extracts flavonoids, saponins, and phenolics); this allows for the possibility of various applications for this co-product.

### 3.3. Quantitative Phytochemical Screening

Table 1 summarizes the total phenolic content in mg gallic acid equivalent (GAE)/100 g dry weight (DW) and the total flavonoid content in mg quercetin equivalent (QE)/100 g DW of each extract. According to the results, the ethanol–water extract had the highest yield and contained the most total phenolic compounds (421.95 mg of GAE/100 g DW) and flavonoids (188.04 mg of QE/100 g DW). Although the extracts obtained with ethyl acetate and acetone contained the same amount of phenolic compounds (126 mg of GAE/100 g DW), the acetone extract contained more flavonoids (42.96 mg QE/100 g DW). Certainly, because of its lipophilic nature, cyclohexane extract contained none of these compounds, whereas chloroform extract contained a lower amount of flavonoids.

This sequential extraction method of increasing solvent polarity allowed for the extraction of all soluble chemical compounds from this *Citrus* species peel. Chloroform and ethyl acetate extracts are lipophilic, whereas acetone and the ethanol–water mixture extracts are hydrophilic. Certainly, the qualitative and quantitative phenolic and flavonoid compositions extracted with these two solvents are dissimilar.

### 3.4. GC-MS Analysis

**Figure 3:**
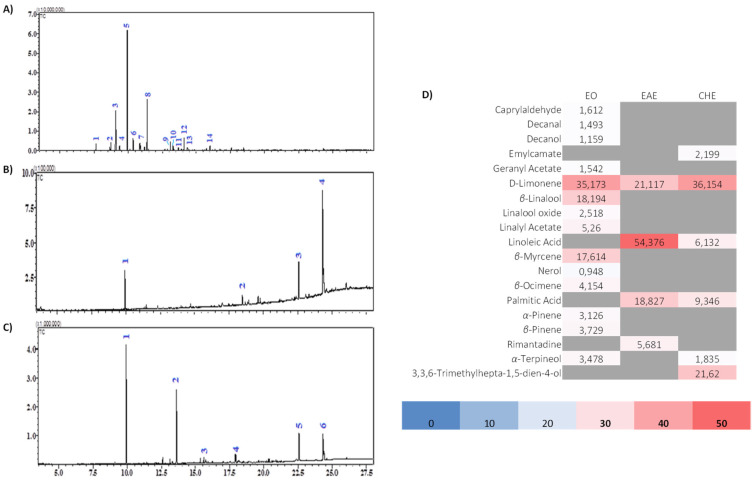
GC-MS chromatogram of the extracts from the peel of *C. aurantium* L.: (**A**) essential oil (EO), (**B**) ethyl acetate, (**C**) cyclohexane, and (**D**) relative quantification (%) of individual compounds identified in each extract. For numbers appearing in (**A**–**C**): please refer to Tables S1–S3 respectively.

Three extracts could be analyzed by GC-MS due to the physicochemical properties of the solvents: essential oil, ethyl acetate, and cyclohexane extracts. Figure 3 depicts chromatograms, and Tables S1–S3 assign chemical compound structures to the corresponding chromatograms.

The EO of *C. aurantium* peel contained 14 major volatile compounds. The results show that monoterpene hydrocarbons (63.80%) predominated in essential oil, while D-limonene (35.17%), β-myrcene (17.61%), and β-linalool (18.19%) were the major constituents. Monoterpene acetate was also present, along with linalyl acetate (5.26%), geranyl acetate (1.54%), and other compounds with lower yields. In previous similar studies on the chemical composition of essential oil (EO) from *C. aurantium* peel, D-limonene has always been the main component with some difference in percentages [7,31,32]: from Western Morocco, researchers found 90.9% [33], from Iran 94.81% [34], and from Brazil 98.66% [31].

Another study reported that Bulgarian essential oil of sour orange peel consists essentially of D-limonene (85.22%), β-myrcene (4.30%), α-pinene (1.29%), and β-linalool (0.42%) [32]. However, the EO from Algeria [35] is strikingly characterized by its high content of β-linalool (12%), followed by trans-carveol (11.9%), *cis*-linalool oxide (8.1%), carvone (5.8%), and D-limonene (2.5%).

With a slight difference in percentages, our findings are similar to those of all previous recent studies. The observed variation could be attributed to the nature of the soil, as well as the geographical and climatic conditions under which the species was grown. It is necessary to specify the harvest season, the maturity of the fruits, and the method of extraction used, as these are determining factors in the quantity and chemical composition of the extracts. Indeed, the alcohols and oxides found in extracts are the result of the degradation of monoterpene hydrocarbons caused by the maturation of the fruit or the heat used during the extraction process. GC-MS analysis of the ethyl acetate extract obtained from the peel led to the identification of four volatile compounds (100%), while that of the cyclohexane extract revealed the presence of six volatile compounds (77.29%).

Both studied extracts contained a mixture of monoterpenes and fatty acids. The main component found in ethyl acetate extract was linoleic acid (54.35%), followed by palmitic acid (18.83%), D-limonene (21.12%), and rimantadine (5.68%), which is a cyclic amine with underlined antiviral activity. In the extract of cyclohexane, D-limonene (36.15%) seemed to be the major constituent, followed by 3,3,6-trimethylhepta-1,5-dien-4-ol (aka *Artemisia* alcohol) (21.62%), palmitic acid (9.35%), linoleic acid (6.13%), emylcamate (2.20%), and α-terpineol (1.83%).

The results for these two extracts are consistent with earlier research on *Citrus* species of different types. Indeed, the main compounds found [36] in the hexane extract of Mexican *C. aurantifolia* are 5,7-dimethoxycoumarin (15.80%), palmitic acid (6.89%), α-terpineol (5.97%), and linoleic acid (0.96%). However, in ethyl acetate and hexane extracts, the Indonesian C. aurantifolia peel contains d-limonene, palmitic acid, α-tocopherol, and linoleic acid [37]. In contrast, D-limonene (31.64%) was found in ethyl acetate extract from the peel of Thai *Citrus hystrix* as the main compound, followed by citronellal (25.99%) and β-pinene (6.83%) [38].

### 3.5. HPLC-DAD Analysis

**Figure 4:**
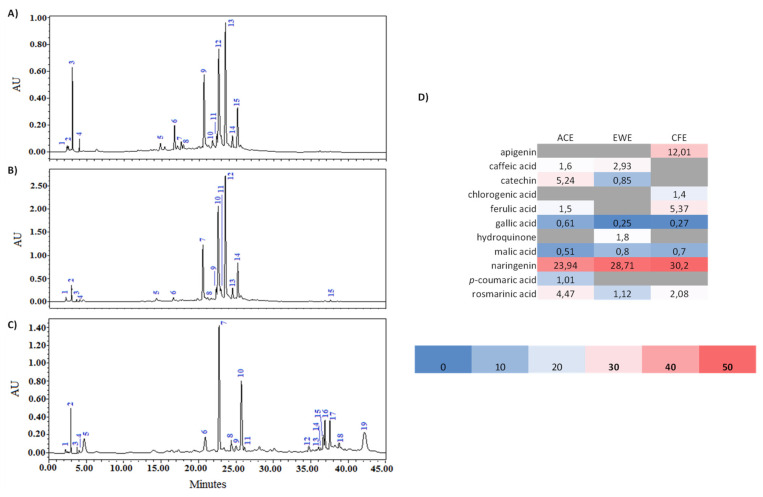
HPLC chromatogram patterns of extracts from *C. aurantium* L. peel: (**A**) acetone ((2) malic acid (0.51%), (3) rosmarinic acid (4.47%), (4) gallic acid (0.61%), (5) caffeic acid (1.60%), (6) catechin (5.24%), (8) p-coumaric acid (1.01%), (10) ferulic acid (1.50%), (12) naringenin (23.94%)); (**B**) ethanol–water ((1) malic acid (0.80%), (2) rosmarinic acid (1.12%), (3) gallic acid (0.25%), (4) hydroquinone (1.80%), (5) caffeic acid (2.93%), (6) catechin (0.85%), (10) naringenin (28.71%)); (**C**) chloroform ((1) malic acid (0.70%), (2) rosmarinic acid (2.08%), (3) gallic acid (0.27%), (6) ferulic acid (5.37%), (7) naringenin (30.20%), (9) chlorogenic acid (1.40%), (19) apigenin (12.01%)); and (**D**) relative quantification (%) of individual compounds identified in each extract.

Figure 4 shows the chromatograms of the acetone (ACE), water–ethanol mixture (EWE), and chloroform (CFE) extracts, while Figure S2 shows the chemical structures of the bioactive fractions found in these three extracts.

The chemical components included in *Citrus* peel extracts were characterized using high-performance liquid chromatography coupled with a diode array detector (HPLC-DAD) by comparing their retention periods and UV spectra to those of authentic standards.

This led to the identification of several chemical compounds: phenolic acids (rosmarinic, gallic, caffeic, chlorogenic, coumaric and ferulic acids), flavonoids (catechin, apigenin, naringenin), hydroquinone, and malic acid. Naringenin, an aglycone of a flavanone found principally in *Citrus* fruits [39], is the main component in all analyzed extracts.

Our results are in agreement with those reported in previous studies that are presented in Table S4. The authors confirmed the presence of naringenin, heperidin, naringin, neohesperidin, tangeritin, apigenin, and catechin, which were considered the main identified flavonoids in *C. aurantium* peel with a difference in percentages, while ferulic, caffeic, p-coumaric, and gallic acids were phenolic acids characteristic of bitter orange peel. In addition, chlorogenic acid and rosmarinic acid were also identified in the methanolic extract of *C. aurantium* peel from Tunisia [40]; furthermore, and similar to our results, three flavonoids were found to be the main bioactive compounds in the hydroethanolic extract of sour orange peel from China [41]: 8 mg/100 g peel naringin, 27 mg/100 g peel naringenin, and 3 mg/100 g peel hesperetin.

### 3.6. Inhibitory Activities of Citrus aurantium Peel Extracts against Intestinal α-Glucosidase and Pancreatic α-Amylase

Essential enzymes for carbohydrate digestion and absorption, intestinal α-glucosidase (EC3.2.1.20) and pancreatic α-amylase (EC3.2.1.1), are described as effective therapeutic targets for modifying the pathologic postprandial hyperglycemia found in T2DM patients. After a preliminary screening, the potential in vitro inhibitory effect of each *Citrus* peel extract was assessed at two different concentrations.

**Figure 5:**
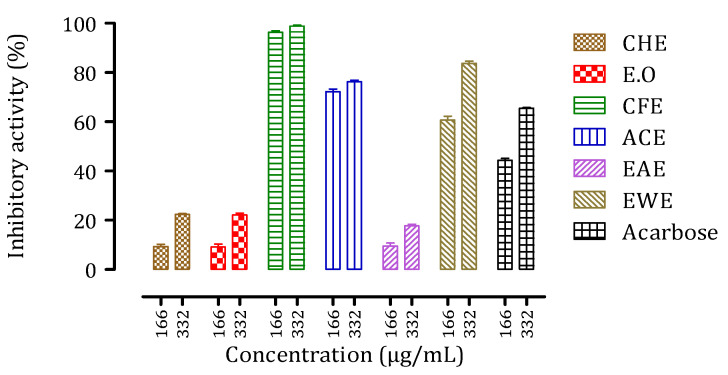
Inhibitory activity of *C. aurantium* peel extracts and Acarbose against intestinal α-glucosidase in vitro for two sample concentrations, 166 µg/mL and 332 µg/mL.

The antidiabetic activity of intestinal α-glucosidase was investigated in vitro at two concentrations (166 g/mL and 332 g/mL) of samples from the peel of *C. aurantium*, with the results shown in Figure 5. The extracts EWE, ACE, and CFE all inhibited α-glucosidase activity in the same way as the control drug (Acarbose). The activity of the other three samples CHE, EO, and EAE was lower than that of the reference. CFE appeared to be the most potent of all the extracts, with an inhibitory activity of over 100% at 332 g/mL. We observed that the effect of increasing extract concentration is significantly more noticeable for less active extracts than for more active extracts.

**Figure 6:**
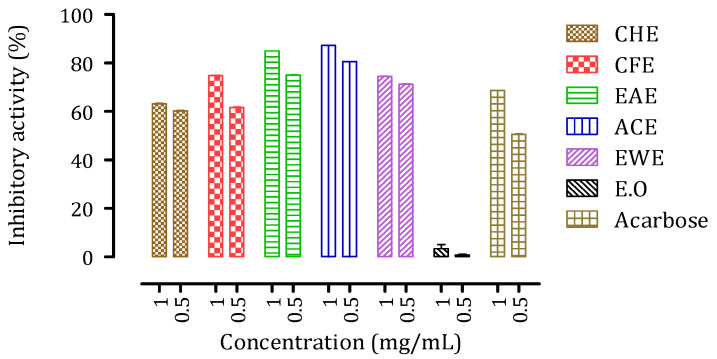
Inhibitory activity of *C. aurantium* peel extracts and Acarbose against pancreatic α-amylase in vitro for two extract concentrations, 0.5 mg/mL and 1 mg/mL.

The assays for pancreatic α-amylase were performed at two concentrations (0.5 mg/mL and 1 mg/mL). As shown in Figure 6, the assays confirmed the inhibitory capacity of *Citrus* peel extracts in vitro. Except for the essential oil (EO), which showed almost no activity even at the highest concentration, all of the extracts studied showed remarkable inhibitory activity (ranging from 60% to 90%). Furthermore, these *Citrus* peel extracts showed higher inhibition capacity than that of Acarbose. Surprisingly, the concentration of the extracts had only little effect on the inhibitory activity against this enzyme.

Reduced postprandial hyperglycemia is one of the therapeutic approaches used to control and treat diabetes mellitus. This is accomplished by inhibiting two major enzymes found in the human digestive tract: α-amylase, which catalyzes the hydrolysis of polysaccharides to broken down oligosaccharides, and α-glucosidase, which catalyzes the hydrolysis of oligosaccharides to monosaccharides (simpler sugars) [42,43]. Currently, the pharmaceutical sector provides various antidiabetic medications to treat diabetic patients, but their side effects are exceedingly hazardous. To address this issue, we have turned to natural resources and herbal therapies that naturally metabolize sugars without causing negative effects [12,13,14,15,16].

The potential antidiabetic efficacy of different extracts from the peel of *C. aurantium* was revealed in this study by examining their inhibitory effects on α-amylase and α-glucosidase in vitro. The extracts tested had strong inhibitory action against both enzymes or a particular enzyme. Some extracts had an activity that was higher than that of the reference molecule Acarbose, while others had an activity that was low or even nonexistent.

For both enzymes, CFE containing a high amount of phenolics and flavonoids, including naringenin and gallic acid, showed high inhibitory activity. The activity of ACE and EWE also containing these phenolics and flavonoids, in a different amount, also showed higher inhibition than Acarbose. Phenolics and flavonoids are natural antidiabetic medicines that inhibit the digesting enzyme and, as a result, lower postprandial blood sugar levels [44]. In this study, correlation analysis showed a significant positive correlation with naringenin and α-glucosidase inhibition (Pearson coefficient correlation (PCC) = 0.884, *p* = 0.017), as well as gallic acid and α-amylase inhibition (PCC = 0.833, *p* = 0.014). In our previous work, we have already demonstrated an interaction between gallic acid and α-amylase [16]. Here, we confirmed that naringenin has a higher affinity (ΔG = −7.2 kcal/mol) for interacting with α-glucosidase in its active site (Figure S3). This result is consistent with prior research that suggested naringenin may have antidiabetic properties [45,46,47]. Naringenin, alongside naringin, both of which are abundant in the peel of *Citrus* species, shows high antidiabetic activity in type 2 diabetic rats induced by NA/STZ [17]. In the light of the present results, we can propose that naringenin could be responsible for the possible antidiabetic activity of *C. aurantium* peel extracts.

### 3.7. Acute Toxicity Evaluation

For a short and extended length of time (48 h–14 days), oral administration of the EO and all extracts from bitter orange peel at a concentration of 2000 mg/kg did not elicit any clinical symptoms of acute toxicity or mortality in any of the mice. Furthermore, during the monitoring period, there was no change in food intake, behavior, or body weight (14 days). Those findings are in congruence with a study carried out in India [48], where it was reported that the oral administration of sour orange hydroalcoholic and aqueous peel extracts at different doses does not lead to any mortality of the animals or any other signs of acute toxicity; moreover, the extracts were considered to be safe up to the dose of 5000 mg/kg. Another study onregarding the administration of Brasilian EO of *C. aurantium* peel [31] did not induce any changes in body weight or toxicity in mice; however, the treatment with EO at 10 mg/kg highly reduced the serum total cholesterol.

## 4. Conclusions

In addition to being a source of D-limonene-rich essential oil, *Citrus aurantium* by-product processing could be a substantial source of phenolic compounds due to the large volume of peel produced. *C. aurantium* fruit residues, which are typically discarded as waste, could be used to produce nutraceuticals. Indeed, due to their good joint inhibitory activities against intestinal α-glucosidase and pancreatic α-amylase, only three extracts from *C. aurantium* peel extracts (chloroform, acetone, and aqueous ethanol), which are rich in phenolics and flavonoids, have significant antidiabetic potential and can manage postprandial hyperglycemia. The presence of naringenin (inhibition of α-glucosidase) and gallic acid (inhibition of α-amylase) in these extracts could explain this activity. Furthermore, unlike manufactured medications, these extracts are regarded as non-toxic inhibitors with no side effects; therefore, they could be used as preventative oral hypoglycemic treatments.

## Figures and Tables

**Table 1:** Yield and quantitative phytochemical screening of *C. aurantium* peel extracts.

Solvent Extract	Yield (%)	Total Phenol Content *	Total Flavonoid Content **
Cyclohexane (CHE)	3.56	–	–
Chloroform (CFE)	3.85	–	18.53 ± 0.67
Ethyl acetate (EAE)	2.40	126.46 ± 1.41	9.72 ± 0.21
Acetone (ACE)	4.45	125.54 ± 1.92	42.96 ± 1.86
Ethanol-water (EWE)	32.10	421.95 ± 5.24	188.04 ± 1.91

* in mg gallic acid equivalent (GAE) per 100 g of dry matter; ** in mg quercetin equivalent (QE) per 100 g of dry matter.

## Data Availability

All the data supporting the findings of this study are included in this article.

## Supplementary Materials

The following are available online at https://www.mdpi.com/article/10.3390/biom11111555/s1, Figure S1: Quercetin (flavonoid) and gallic acid (polyphenol) calibration curves, Figure S2: Chemical structures of the molecules identified in *C. aurantium* L peel extracts: chloroform extract (CFE), acetone extract (ACE), and hydro-alcoholic extract (EWE), Figure S3: Molecular docking of naringenin on human α-glucosidase active site; Table S1: Chemical constituents of the essential oil of *C. aurantium* L. peel, Table S2: Chemical composition of ethyl acetate extract from the peel of *C.* *aurantium* L., Table S3: Chemical composition of cyclohexane extract from the peel of *C. aurantium* L., Table S4: The main phenolic compounds identified in *C. aurantium* L. peel and in *C. aurantium* L var. amara reported in the literature.

## References

[1] Reuther W., Batchelor L.D., Webber H.J. (1967). The Citrus Industry. Vol. I..

[2] Cerdagne I. (2004). L’oranger amer: Citrus aurantium var.amara link. Diss Thése Dr. Pharm..

[3] Sharma K., Mahato N., Cho M.H., Lee Y.R. (2017). Converting citrus wastes into value-added products: Economic and environmently friendly approaches. Nutrition.

[4] Suntar I., Khan H., Patel S., Celano R., Rastrelli L. (2018). An overview on Citrus aurantium L.: Its functions as food ingredient and therapeutic agent. Oxidative Med. Cell. Longev..

[5] Güven C., Taşkin E., Kaya S.T., Sevgiler Y. (2018). The potential anti-diabetic effects of some plant species. Nat. Eng. Sci..

[6] Khan H., Nabavi S.M., Sureda A., Mehterov N., Gulei D., Berindan-Neagoe I., Taniguchi H., Atanasov A.G. (2018). Therapeutic potential of songorine, a diterpenoid alkaloid of the genus Aconitum. Eur. J. Med. Chem..

[7] Bendaha H., Bouchal B., El Mounsi I., Salhi A., Berrabeh M., El Bellaoui M., Mimouni M. (2016). Chemical composition, antioxidant, antibacterial and antifungal activities of peel essential oils of citrus aurantium grown in Eastern Morocco. Der Pharm. Lett..

[8] Bendaha H., Mimouni M., Karrouchi K., El Mounsi I., Bouchal B. (2016). Byproducts evaluation: Phytochemical investigation and byproducts evaluation: Phytochemical investigation and antioxidant activity of extracts of Eastern Moroccan (Oujda) Citrus. Rev. Microbiol. Ind. Sanit. Et Environn..

[9] Mahato N., Sinha M., Sharma K., Koteswararao R., Cho M.H. (2019). Modern extraction and purification techniques for obtaining high purity food-grade bioactive compounds and value-added co-products from Citrus wastes. Foods.

[10] Suryawanshi J.A.S. (2011). An overview of Citrus aurantium used in treatment of various diseases. Afr. J. Plant. Sci..

[11] Saunders A., Messer L.H., Forlenza G.P. (2019). MiniMed 670G hybrid closed loop artificial pancreas system for the treatment of type 1 diabetes mellitus: Overview of its safety and efficacy. Expert Rev. Med. Devices.

[12] Hano C., Renouard S., Molinié R., Corbin C., Barakzoy E., Doussot J., Lamblin F., Lainé E. (2013). Flaxseed (Linum usitatissimum L.) extract as well as (+)-secoisolariciresinol diglucoside and its mammalian derivatives are potent inhibitors of α-amylase activity. Bioorganic. Med. Chem. Lett..

[13] Ullah M.A., Tungmunnithum D., Garros L., Drouet S., Hano C., Abbasi B.H. (2019). Effect of ultraviolet-C radiation and melatonin stress on biosynthesis of antioxidant and antidiabetic metabolites produced in in vitro callus cultures of lepidium sativum L.. Int. J. Mol. Sci..

[14] Ullah M.A., Tungmunnithum D., Garros L., Hano C., Abbasi B.H. (2019). Monochromatic lights-induced trends in antioxidant and antidiabetic polyphenol accumulation in in vitro callus cultures of Lepidium sativum L.. J. Photochem. Photobiol. B Biol..

[15] Khurshid R., Ullah M.A., Tungmunnithum D., Drouet S., Shah M., Zaeem A., Hameed S., Hano C., Abbasi B.H. (2020). Lights triggered differential accumulation of antioxidant and antidiabetic secondary metabolites in callus culture of Eclipta alba L.. PLoS ONE.

[16] Tiji S., Bouhrim M., Addi M., Drouet S., Lorenzo J.M., Hano C., Bnouham M., Mimouni M. (2021). Linking the phytochemicals and the α-glucosidase and α-amylase enzyme inhibitory effects of nigella sativa seed extracts salima. Foods.

[17] Ahmed O.M., Hassan M.A., Abdel-Twab S.M., Abdel Azeem M.N. (2017). Navel orange peel hydroethanolic extract, naringin and naringenin have anti-diabetic potentials in type 2 diabetic rats. Biomed. Pharmacother..

[18] Alu’Datt M.H., Rababah T., Alhamad M.N., Al-Mahasneh M.A., Ereifej K., Al-Karaki G., Al-Duais M., Andrade J.E., Tranchant C.C., Kubow S. (2017). Profiles of free and bound phenolics extracted from: Citrus fruits and their roles in biological systems: Content, and antioxidant, anti-diabetic and anti-hypertensive properties. Food Funct..

[19] Jaradat N., Hussen F., Ali A.A. (2015). Preliminary phytochemical screening, quantitative estimation of total flavonoids, total phenols and antioxidant activity of Ephedra alata decne. J. Mater. Environ. Sci..

[20] Alilou H., Bencharki B., Mina L., Hassani I. (2014). Screening phytochimique et identification spectroscopique des flavonoïdes d’ Asteriscusgraveolenssubsp. odorus. Afr. Sci. Rev. Int. Des. Sci. Et Technol..

[21] Li B.B., Smith B., Hossain M.M. (2006). Extraction of phenolics from citrus peels: I. Solvent extraction method. Sep. Purif. Technol..

[22] Tiji S., Benayad O., Berrabah M., El Mounsi I., Mimouni M. (2021). Phytochemical profile and antioxidant activity of Nigella sativa L growing in Morocco. Sci. World J..

[23] Campos M.D.G., Markham K.R. (2007). Structure Information from HPLC and On-Line Measured Absorption Spectra: Flavones, Flavonols and Phenolic Acids.

[24] Abid S., Lekchiri A., Mekhfi H., Ziyyat A., Legssyer A., Aziz M., Bnouham M. (2014). Inhibition of α-glucosidase and glucose intestinal absorption by Thymelaea hirsuta fractions. J. Diabetes.

[25] Juvekar A.R., Khatri D.K. (2014). α-Glucosidase and α-amylase inhibitory activity of Indigofera cordifolia seeds and leaves extract. Int. J. Pharm. Pharm. Sci..

[26] Daoudi N.E., Bouhrim M., Ouassou H., Legssyer A., Mekhfi H., Ziyyat A., Aziz M., Bnouham M. (2020). Inhibitory effect of roasted/unroasted Argania spinosa seeds oil on α-glucosidase, α-amylase and intestinal glucose absorption activities. South Afr. J. Bot..

[27] Tchoumtchoua J., Mouchili O.R., Ateba S.B., Zingue S., Halabalaki M., Mbanya J.C., Skaltsounis A.L., Njamen D. (2014). Safety assessment of the methanol extract of the stem bark of Amphimas pterocarpoides harms: Acute and subchronic oral toxicity studies in Wistar rats. Toxicol. Rep..

[28] Terry P., Giovannucci E., Michels K.B., Bergkvist L., Hansen H., Holmberg L., Wolk A. (2001). Fruit, vegetables, dietary fiber, and risk of colorectal cancer. J. Natl. Cancer Inst..

[29] R S., Gurunathan J. (2020). Metabolites from the citrus extracts inhibit the activity of selected proteins in Indian Cobra (Naja naja) venom. J. Ethnopharmacol..

[30] Gunwantrao B.B., Bhausaheb S.K., Ramrao B.S., Subhash K.S. (2016). Antimicrobial activity and phytochemical analysis of orange (Citrus aurantium L.) and pineapple (Ananas comosus (L.) Merr.) peel extract. Ann. Phytomedicine.

[31] Costa C.A.R.A., Cury T.C., Cassettari B.O., Takahira R.K., Flório J.C., Costa M. (2013). Citrus aurantium L. essential oil exhibits anxiolytic-like activity mediated by 5-HT1A-receptors and reduces cholesterol after repeated oral treatment. BMC Complementary Altern. Med..

[32] Teneva D., Denkova-Kostova R., Goranov B., Hristova-Ivanova Y., Slavchev A., Denkova Z., Kostov G. (2019). Chemical composition, antioxidant activity and antimicrobial activity of essential oil from Citrus aurantium L zest against some pathogenic microorganisms. Z. Fur Nat.-Sect. C J. Biosci..

[33] Essadik F.Z., Haida S., Kribii A., Kribii A.R., Ounine K. (2015). Antioxidant activity of Citrus aurantium L. var. amara Peel from western of Morocco, identification of volatile compounds of its essential oil by GC-MS and a preliminary study of their antibacterial activity. Int. J. Innov. Sci. Res..

[34] Sanei-Dehkordi A., Sedaghat M.M., Vatandoost H., Abai M.R. (2016). Chemical compositions of the peel essential oil of Citrus aurantium and its natural larvicidal activity against the malaria vector Anopheles stephensi (Diptera: Culicidae) in comparison with Citrus paradisi. J. Arthropod-Borne Dis..

[35] Abderrezak M.K., Abaza I., Aburjai T., Kabouche A., Kabouche Z. (2014). Comparative compositions of essential oils of Citrus aurantium growing in different soils. J. Mater. Environ. Sci..

[36] Sandoval-Montemayor N.E., García A., Elizondo-Treviño E., Garza-González E., Alvarez L., Del Rayo Camacho-Corona M. (2012). Chemical composition of hexane extract of Citrus aurantifolia and anti-Mycobacterium tuberculosis activity of some of its constituents. Molecules.

[37] Asmah N., Suniarti D., Margono A., Mas’ud Z., Bachtiar E. (2020). Identification of active compounds in ethyl acetate, chloroform, and N-hexane extracts from peels of Citrus aurantifolia from Maribaya, West Java, Indonesia. J. Adv. Pharm. Technol. Res..

[38] Chanthaphon S., Chanthachum S., Hongpattarakere T. (2008). Antimicrobial activities of essential oils and crude extracts from tropical Citrus spp. Against food-related microorganisms. Songklanakarin J. Sci. Technol..

[39] Hernández-Aquino E., Muriel P. (2017). Naringenin and the liver. Liver Pathophysiology: Therapies and Antioxidants.

[40] Jabri Karoui I., Marzouk B. (2013). Characterization of bioactive compounds in Tunisian bitter orange (Citrus aurantium L.) peel and juice and determination of their antioxidant activities. BioMed Res. Int..

[41] Zhang L., Xu X., Jiang T., Wu K., Ding C., Liu Z., Zhang X., Yu T., Song C. (2018). Citrus aurantium naringenin prevents osteosarcoma progression and recurrence in the patients who underwent osteosarcoma surgery by improving antioxidant capability. Oxidative Med. Cell. Longev..

[42] Arumugam G., Manjula P., Paari N. (2013). A review: Anti diabetic medicinal plants used for diabetes mellitus. J. Acute Dis..

[43] Duarte A.M., Guarino M.P., Barroso S., Gil M.M. (2020). Phytopharmacological strategies in the management of type 2 diabetes mellitus. Foods.

[44] Kamtekar S., Keer V., Patil V. (2014). Estimation of phenolic content, flavonoid content, antioxidant and alpha amylase inhibitory activity of marketed polyherbal formulation. J. Appl. Pharm. Sci..

[45] Alam M.A., Subhan N., Rahman M.M., Uddin S.J., Reza H.M., Sarker S.D. (2014). Effect of Citrus flavonoids, naringin and naringenin, on metabolic syndrome and their mechanisms of action. Adv. Nutr..

[46] Sahnoun M., Trabelsi S., Bejar S. (2017). Citrus flavonoids collectively dominate the α-amylase and α-glucosidase inhibitions. Biologia.

[47] Zhang K., Ding Z., Duan W., Mo M., Su Z., Bi Y., Kong F. (2020). Optimized preparation process for naringenin and evaluation of its antioxidant and α-glucosidase inhibitory activities. J. Food Process. Preserv..

[48] Sharma M., Fernandes J., Ahirwar D., Jain R. (2008). Hypoglycemic and hypolipidimic activity of alcoholic extract of citrus aurantium in normal and alloxan-induced diabetic rats. Pharmacologyonline.

