# Supplementary material for: Phytochemical Profile, α-Glucosidase, and α-Amylase Inhibition Potential and Toxicity Evaluation of Extracts from Citrus aurantium (L) Peel, a Valuable By-Product from Northeastern Morocco
Source: Biomolecules. 2021 Oct 20;11(11):1555. doi: 10.3390/biom11111555 (PMC8615658; doi:10.3390/biom11111555)
Supplement: Supplementary file 1 [file biomolecules-11-01555-s001.zip › biomolecules-1417878-supplementary.pdf]

Phytochemical Profile, Inhibition Potential Toward α-Glucosidase and α-Amylase Activities and Toxicity Evaluation of Peel Extracts of *Citrus aurantium* (L) from Northeastern Morocco

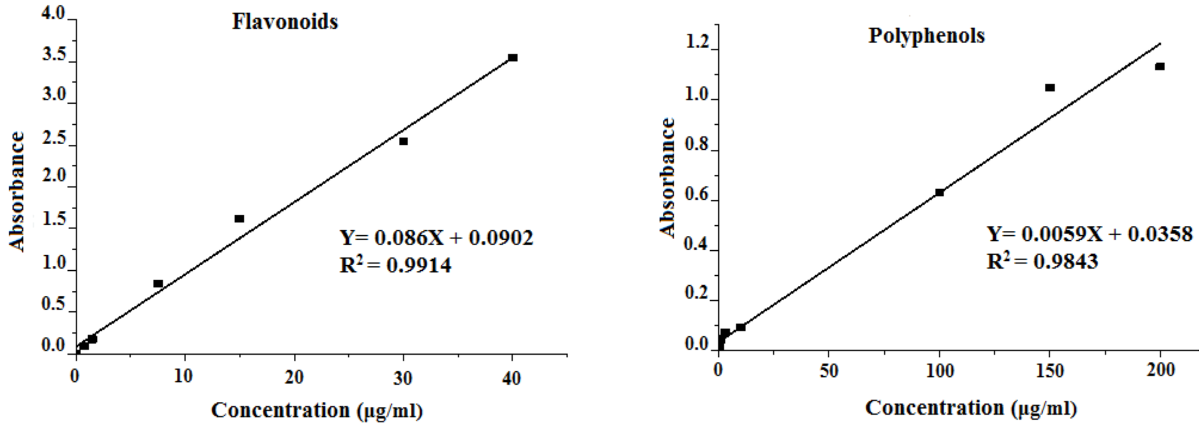

Figure S1: Quercetin (Flavonoids) and Gallic acid (Polyphenols) calibration curves

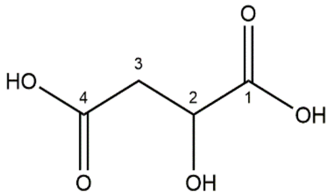

Malic acid

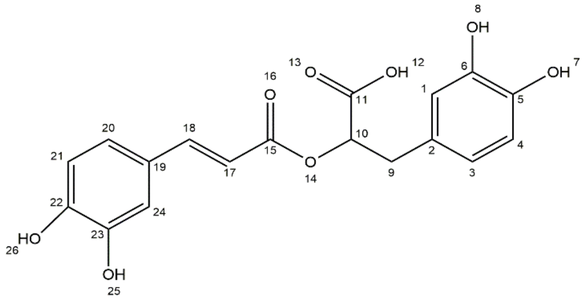

Rosmarinic acid

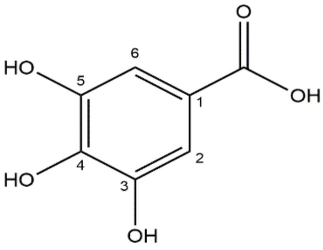

Gallic acid

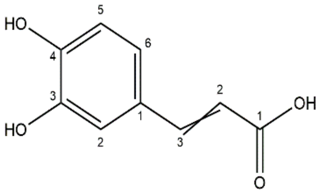

Caffeic acid

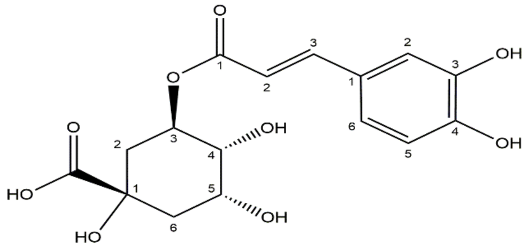

Chlorogenic acid

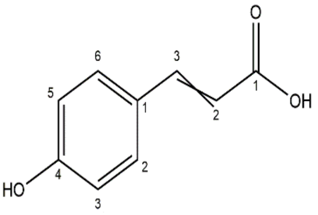

Coumaric acid

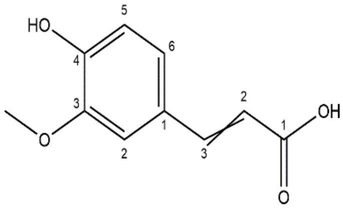

Ferulic acid

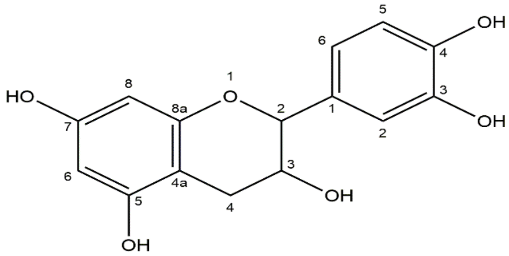

Catechin

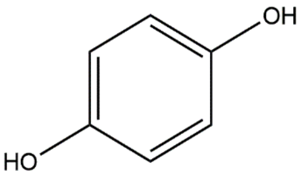

Hydroquinone

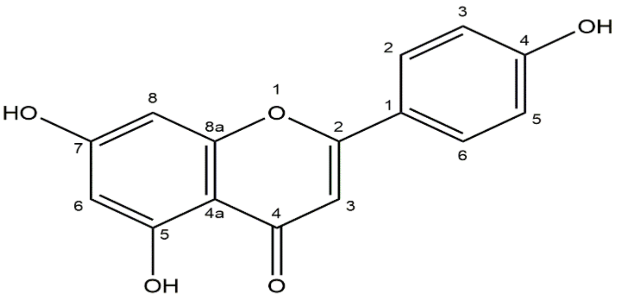

Apigenin

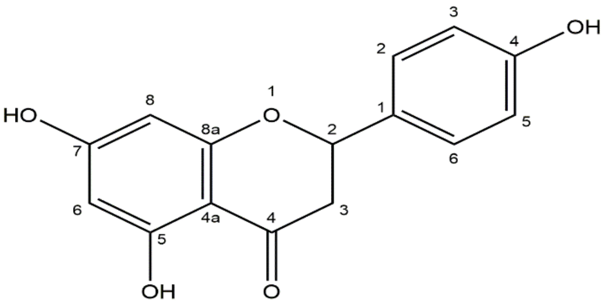

Naringenin

Figure S2: Chemical structures of the molecules identified in *C. aurantium* L peel extracts: chloroform extract (CFE), acetone extract (ACE) and hydro-alcoholic extract (EWE).

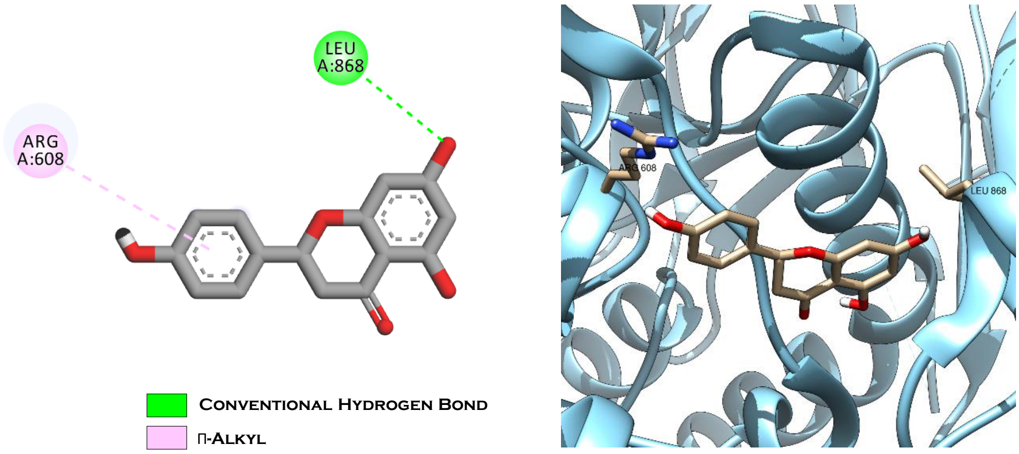

COMPOUNDS	BINDING SCORE (KCAL/MOL)	INTERACTING RESIDUES	INTERACTION TYPE
Naringenin	-7.2	ARG 608, LEU 868	H-bonding, π-Alkyl

Figure S3: Molecular Docking of Naringenin on human α-glucosidase active site.

Table S1: The chemical constituents of the essential oil *C. aurantium* L. peel

Peak Number	Retention time (min)	Peak Area (%)	Molecules Name	Molecules structure
**1**	7.625	3.126	*α*-Pinene	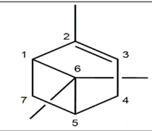
**2**	8.725	3.729	*β*-Pinene	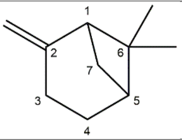
**3**	9.083	17.614	*β*-Myrcene	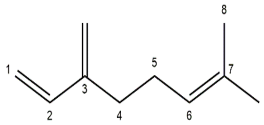
**4**	9.367	1.612	Caprylaldehyde	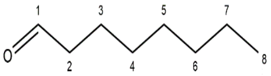
**5**	9.942	35.173	D-Limonene	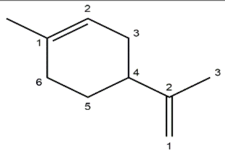
**6**	10.358	4.154	*β*-Ocimene	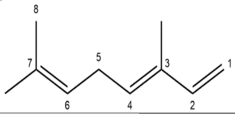
**7**	10.875	2.518	Linalool oxide	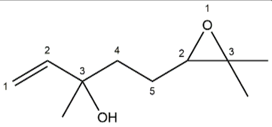
**8**	11.392	18.194	*β*-Linalool	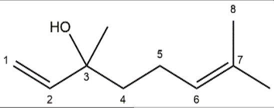
**9**	13.092	3.478	*α*-Terpineol	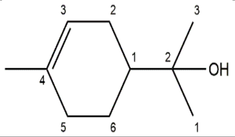
**10**	13.283	1.493	Decanal	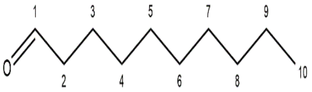
**11**	13.675	0.948	Nerol	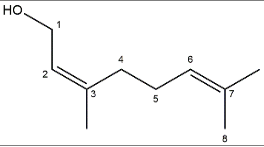
**12**	14.092	5.260	Linalyl Acetate	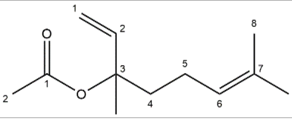
**13**	14.342	1.159	Decanol	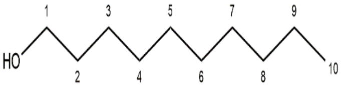

**14**	16.000	1.542	Geranyl Acetate	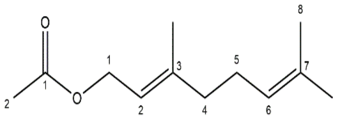

Table S2: Chemical composition of ethyl acetate extract from the peel of *C. aurantium* L.

Peak Number	Retention time (min)	Peak Area (%)	Molecules Name	Molecules structure
**1**	9.933	21.117	D-Limonene	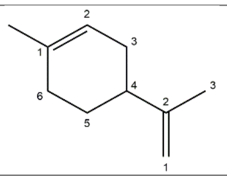
**2**	18.475	5.681	Rimantadine	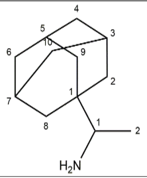
**3**	22.583	18.827	Palmitic Acid	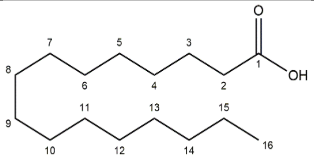
**4**	24.325	54.376	Linoleic Acid	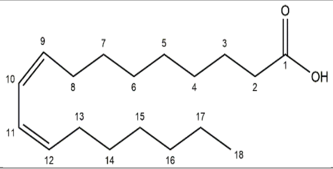

Table S3: The chemical composition of cyclohexane extract from the peel of *C. aurantium* L.

Peak Number	Retention time (in)	Peak Area (%)	Names Molecules	Molecules structure
**1**	9.933	36.154	D-Limonene	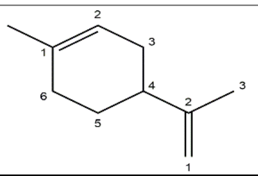
**2**	13.583	21.620	1,5-Heptadien-4-ol, 3,3,6-trimethyl-	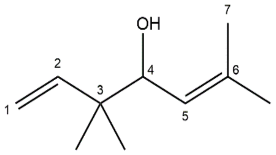
**3**	15.558	1.835	α-Terpineol	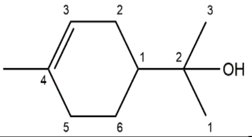
**4**	17.917	2.199	Emylcamate	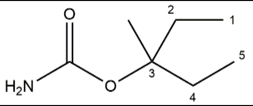
**5**	22.575	9.346	Palmitic Acid	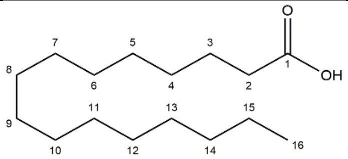
**6**	24.325	6.132	Linoleic Acid	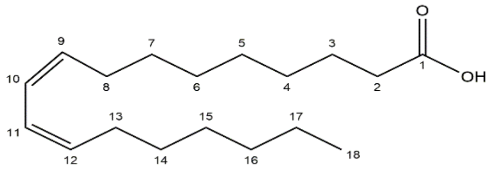

Table S4: The main phenolic compounds identified in *C. aurantium* L. peel and in *C. aurantium L* var. *amara* reported in the literature.

Species name	Fruit's origin	Extract	Identified Phenolic components	References
Bitter orange (*Citrus aurantium*. L)	USA	80% Ethanol	Isonaringin, Naringin, Hesperidin, Neohesperidin, Naringenin, Hesperitin, Nobiletin and Tangeritin	[33]
	Italy	Methanol	Caffeic, p-Coumaric, Ferulic, and Synapic acids, Naringin, Neohesperidin, Hesperidin, Narirutin, and Neoericitrin	[34]
	Iran	Methanol	Gallic, Caffeic, and Syringic acids, Pyrogallol, Naringin, Quercetin, and Rutin	[35]
	Tunisia	Methanol	Gallic, Chlorogenic, Rosmarinic, Trans-2-hydroxycinnamic, p-Coumaric, Ferulic acids, Epicatechin, Catechin, Naringin and Rutin	[31]
	China	20% Ethanol	Naringin, Hesperidin, Neohesperidin, Naringenin, Hesperitin, Narirutin, and Neoericitrin	[32]
Bitter orange (*Citrus aurantium L* var. *amara*)	Italy	80% Ethanol		[36]
	Cuba	70% Ethanol	Apigenin-6,8-di-C-glucoside, Diosmetin-6,8-di-C-glucoside, Rhoifolin 4′-glucoside, Neoeriocitrin, Naringin Neohesperidin, Rhoifolin, Neodiosmin, Brutieridin, Nobiletin	[37]
	China	80% Methanol	Tangeretin, Auraptene, Nobiletin, Scopoletin, Hesperitin-O-rhamnoside, Diosmetin-O-glucoside, Apigenin, Hesperidin, Naringenin-O-glucoside, Naringenin, Asparagine, Luteolin-O-glucoside, Eriocitrin, Narirutin, Naringin, Neohesperidin	[38]
